# Application of artificial intelligence in medical education: Saudi Arabian perspective

**DOI:** 10.3389/fmed.2026.1779605

**Published:** 2026-03-26

**Authors:** Hosam Al-Jehani, Ahmed Hafez Mousa, Sadeem Albulaihed

**Affiliations:** 1 Department of Neurosurgery, King Fahd Hospital of the University, Al Khobar, Saudi Arabia; 2 Department of Neurology and Neurosurgery, Montreal Neurological Institute and Hospital, McGill University, Montreal, QC, Canada; 3 Department of Neurosurgery, Houston Methodist Hospital, Weill Cornell University, Houston, TX, United States; 4 Department of Neurosurgery, Imam Abdulrahman Bin Faisal University, Al Khobar, Saudi Arabia; 5 Neurosciences, Dubai Health, Dubai, United Arab Emirates; 6 Department of Neurosurgery, Graduate Medical Education, Mohammed Bin Rashid University of Medicine and Health Sciences, Dubai Health, Dubai, United Arab Emirates

**Keywords:** artificial intelligence, healthcare, machine learning, medical education, Saudi Arabia

## Abstract

**Introduction:**

Artificial intelligence (AI) is increasingly reshaping medical education by enhancing learning efficiency, personalization, and assessment. In Saudi Arabia, national investments in digital health and education under Vision 2030 have created a favorable environment for AI integration. However, the adoption of AI in medical education must be carefully balanced to preserve essential humanistic elements such as empathy, mentorship, and interpersonal communication.

**Methods:**

This narrative review examines the current state of AI applications in medical education in Saudi Arabia. A structured literature search was conducted across peer-reviewed databases and relevant gray literature to identify studies published in English or Arabic within the past 5 years. Data were extracted, critically appraised, and synthesized narratively to highlight key applications, benefits, challenges, and future directions.

**Results:**

AI applications in Saudi Arabian medical education include adaptive learning platforms, simulation-based training, virtual and augmented reality, intelligent tutoring systems, and AI-supported assessment tools. These approaches have been associated with improved learner engagement, knowledge retention, and personalized educational experiences among medical students, residents, and trainees. Despite these advances, formal integration of AI-related competencies—such as data literacy, algorithm interpretation, and ethical AI use—remains limited within current curricula.

**Conclusion:**

Artificial intelligence has significant potential to transform medical education in Saudi Arabia and support the development of a future-ready healthcare workforce. While national initiatives and investments have accelerated AI adoption, further research, faculty development, ethical governance, and curriculum reform are required to ensure responsible, effective, and human-centered implementation

## Background

Artificial intelligence (AI) is a rapidly evolving field with transformative potential across multiple sectors, including healthcare. In medicine, AI applications span diagnostic imaging, drug discovery, personalized treatment planning, and increasingly, medical education (1). In recent years, growing attention has been directed toward AI’s capacity to enhance the quality, efficiency, and accessibility of medical training.

Saudi Arabia has emerged as a regional leader in AI development and implementation, driven largely by the national Vision 2030 initiative, which seeks to diversify the economy and reduce reliance on oil revenues (2). Within this framework, the healthcare sector has actively adopted AI technologies to optimize service delivery, clinical decision-making, and educational processes.

Despite these advances, the application of AI in medical education within Saudi Arabia remains at an early developmental stage. Nevertheless, interest in this domain is increasing, motivated by the need to elevate educational standards, address workforce shortages, and continuously upskill healthcare professionals (3). Although Saudi Arabia has achieved notable progress in healthcare infrastructure and service provision, challenges persist, including limited numbers of trained specialists and the need for scalable, high-quality educational models.

AI-driven educational tools offer innovative solutions to these challenges. Simulation-based platforms can provide immersive, realistic clinical scenarios that allow trainees to practice procedural and decision-making skills in a controlled environment. Similarly, AI-powered virtual assistants can support personalized learning, guide students through coursework, and offer targeted feedback (4). Furthermore, AI-enabled assessment systems may improve the objectivity and precision of learner evaluation, enabling educators to identify individual knowledge gaps and tailor educational interventions accordingly (5).

Despite these advantages, concerns remain regarding the potential erosion of human connection in medical education. Empathy, professionalism, and interpersonal communication are core competencies that cannot be fully replicated by AI systems. Ensuring that AI augments rather than replaces human mentorship is therefore essential (6).

Traditional and online educational models share fundamental instructional elements, including structured curricula, assessments, and collaborative learning. However, e-learning platforms—whether synchrono us or asynchronous—offer increased flexibility and program diversity. While some studies report comparable outcomes between conventional and online education, others suggest differences in engagement and learning preferences (3).

Overall, AI has the potential to significantly enhance medical education and training standards in Saudi Arabia. Although current applications remain limited, the growing interest and national investment in this field position AI as a powerful adjunct to medical education, provided its implementation preserves the humanistic foundations of medical practice.

## Aims and objectives

1. To examine the current status of AI integration in medical education in Saudi Arabia.
2. To identify the potential benefits of AI in medical education, including improvements in learning outcomes, clinical decision-making, and patient care.

## Methodology

This narrative review addresses the current state of AI applications in medical education in Saudi Arabia. A structured search strategy was employed to identify relevant peer-reviewed literature and gray sources. Inclusion criteria encompassed studies published within the last 5 years, focusing on AI applications in medical education in Saudi Arabia, and written in English or Arabic.

Following study selection, data extraction and synthesis were conducted to summarize key findings. The quality and relevance of included studies were critically appraised, and results were synthesized narratively to identify prevailing themes, gaps in knowledge, and future directions.

## Results

Artificial intelligence has been integrated into medical education through multiple approaches, including gamification, virtual and augmented reality, adaptive learning systems, and simulation-based training. These strategies have demonstrated improvements in learner engagement, knowledge retention, and educational satisfaction among medical students, residents, and trainees.

In Saudi Arabia, several initiatives have been launched to incorporate AI into medical education, including the development of AI-based curricula and the adoption of simulation platforms. While the structure of postgraduate medical education varies internationally, curricular content remains largely comparable. Traditionally, medical education has emphasized knowledge acquisition through didactic teaching alongside the development of practical and professional skills.

As AI systems are capable of processing and retaining vast amounts of data, future physicians may rely less on memorization and repetitive procedural practice. Instead, new competencies—such as data literacy, statistics, AI ethics, and algorithm interpretation—will become increasingly important. Clinicians will be required to accurately input data, interpret AI-generated outputs, and communicate AI-supported recommendations effectively to patients. Although national healthcare policies encourage the development of such competencies (2), formal integration into most clinical training programs remains limited (7, 8). Table 1 shows types of AI applications in medical education.

**TABLE 1:** Types of artificial intelligence (AI) applications in medical education (6, 9).

Types of AI applications	Examples	Description
Intelligent tutoring systems	DextER, i-Human, and Touch Surgery	AI systems that provide personalized learning experiences for students based on their individual strengths and weaknesses.
Virtual reality and augmented reality	Microsoft HoloLens, CAE Healthcare’s VimedixAR, and Stanford’s Virtual Human Interaction Lab	Using AI to create immersive simulations of medical scenarios to help students gain practical experience.
Natural language processing (NLP)	IBM Watson, Linguamatics, and Nuance	AI-powered chatbots that can simulate conversations with patients to help students develop communication skills.
Predictive analytics	HBS, techtarget.	Using AI to analyze student data to identify areas where they need extra support or instruction.
Clinical decision support systems (CDSS)	BJSS,BI-RADS, CAD, PUFF	AI systems that assist in making clinical decisions by providing recommendations based on patient data.

## Discussion

The integration of artificial intelligence (AI) into medical education represents a paradigm shift with far-reaching implications for how future physicians are trained, assessed, and supported throughout their professional development. In the Saudi Arabian context, this transformation is particularly relevant, given the nation’s rapid healthcare expansion, strategic investment in digital transformation under Vision 2030, and the growing demand for a highly skilled and adaptable medical workforce. This narrative review highlights both the opportunities and challenges associated with the adoption of AI in medical education and underscores the need for a deliberate, ethically grounded, and pedagogically sound implementation strategy.

### Transformative potential of AI in medical education

Artificial intelligence has the capacity to fundamentally redefine traditional educational models by moving away from standardized, one-size-fits-all curricula toward adaptive, learner-centered frameworks. Through continuous analysis of learner performance data, AI systems can personalize educational content, pacing, and assessment, thereby addressing individual strengths and weaknesses (5). Such personalization is particularly valuable in medical education, where trainees demonstrate wide variability in learning styles, clinical exposure, and skill acquisition rates.

In Saudi Arabia, where medical schools and training programs are expanding to meet workforce demands, AI-driven adaptive learning platforms may help standardize educational quality while accommodating increasing trainee numbers. These systems can support early identification of struggling learners, allowing for targeted remediation and potentially reducing attrition rates. Moreover, AI-supported formative assessment tools provide real-time feedback, which is known to enhance retention, motivation, and long-term learning outcomes (6).

### Simulation, virtual reality, and experiential learning

One of the most impactful applications of AI in medical education lies in simulation-based learning, particularly when combined with virtual reality (VR) and augmented reality (AR). AI-powered simulations allow trainees to engage with complex clinical scenarios that closely mirror real-world practice, including rare or high-risk conditions that may be infrequently encountered during training. These immersive environments enable repetitive practice without patient risk, fostering procedural competence, clinical reasoning, and decision-making under pressure (7).

In the Saudi healthcare system, where patient safety and quality assurance are national priorities, AI-driven simulation platforms may play a crucial role in competency-based education. Such tools can objectively track performance metrics, including response times, error rates, and adherence to clinical guidelines, providing educators with granular insights into trainee progress. Importantly, simulation-based education aligns well with modern accreditation standards that emphasize measurable competencies rather than time-based training alone.

### Redefining the role of medical knowledge and clinical skills

The increasing sophistication of AI systems challenges traditional assumptions about the core competencies required of physicians. As AI algorithms become capable of rapidly synthesizing vast quantities of medical data, the physician’s role is likely to shift from information recall toward higher-order cognitive functions, including critical appraisal, contextual judgment, ethical reasoning, and patient communication.

This evolution has significant implications for medical curricula in Saudi Arabia. While foundational biomedical knowledge remains essential, greater emphasis may be needed on data literacy, statistical reasoning, AI ethics, and human–machine interaction. Clinicians of the future must be able to understand the limitations of AI systems, recognize potential biases, and appropriately integrate algorithmic recommendations into individualized patient care. Currently, however, most medical training programs in Saudi Arabia—and globally—do not formally incorporate these competencies, highlighting an important curricular gap (8).

### Faculty development and educational culture

Successful implementation of AI in medical education is contingent not only on technological infrastructure but also on faculty readiness and institutional culture. Many medical educators may lack formal training in AI concepts, leading to uncertainty or resistance toward adoption. Without adequate faculty development programs, AI tools risk being underutilized or misapplied.

In Saudi Arabia, targeted faculty training initiatives are essential to ensure educators can critically evaluate AI technologies, integrate them meaningfully into curricula, and guide learners in their appropriate use. Institutions must foster a culture in which AI is viewed as an educational enhancer rather than a replacement for human instruction. Faculty engagement is particularly important in maintaining the mentorship, professionalism, and ethical role-modeling that are central to medical education (9).

### Ethical, legal, and social considerations

Ethical considerations represent one of the most significant challenges to the widespread adoption of AI in medical education. Issues such as data privacy, algorithmic bias, transparency, and accountability are especially pertinent in healthcare contexts. AI systems trained on non-representative datasets may perpetuate inequities or reinforce existing biases, potentially affecting both educational assessment and clinical decision-making.

In Saudi Arabia, the ethical deployment of AI must align with national regulations, cultural values, and societal expectations. Clear governance frameworks are needed to define data ownership, consent, and acceptable use of learner and patient data in educational AI systems. Additionally, safeguards must be implemented to prevent over-reliance on AI at the expense of clinical judgment and interpersonal skill development.

### Humanism and the preservation of the doctor–patient relationship

A recurring concern in the literature is the potential erosion of humanism in medicine due to increasing technological dependence. While AI can enhance efficiency and accuracy, it cannot replicate empathy, compassion, or moral reasoning—qualities that are fundamental to effective medical practice. Medical education must therefore ensure that AI integration does not diminish opportunities for trainees to develop communication skills, emotional intelligence, and professional identity.

In the Saudi context, where cultural sensitivity, family-centered care, and strong physician–patient relationships are highly valued, preserving the human dimension of medicine is particularly important. AI should be positioned as a supportive tool that augments clinical care while reinforcing the importance of human connection.

### Alignment with national health and education strategies

Saudi Arabia’s Vision 2030 provides a strategic framework that strongly supports digital innovation, research, and human capital development. AI integration in medical education aligns closely with these national objectives, offering an opportunity to develop a future-ready healthcare workforce capable of leveraging advanced technologies responsibly.

However, achieving this vision requires coordinated efforts among medical schools, healthcare institutions, regulatory bodies, and technology developers. National guidelines for AI education, standardized competency frameworks, and outcome-based evaluation metrics will be essential to ensure consistent and equitable implementation across institutions.

### Future directions and research implications

Despite growing interest, empirical evidence on the long-term effectiveness of AI-based educational interventions in Saudi Arabia remains limited. Future research should focus on comparative effectiveness studies, cost-benefit analyses, and longitudinal outcomes, including clinical performance, patient safety, and professional development. Additionally, qualitative research exploring learner and faculty perceptions will be valuable in understanding barriers to adoption and optimizing user-centered design.

Interdisciplinary collaboration between medical educators, data scientists, ethicists, and policymakers will be critical in shaping AI-enabled educational ecosystems that are innovative, ethical, and culturally appropriate.
